# Anaerobic digestion of commercial PLA and PBAT biodegradable plastic bags: Potential biogas production and ^1^H NMR and ATR-FTIR assessed biodegradation

**DOI:** 10.1016/j.heliyon.2023.e16691

**Published:** 2023-05-26

**Authors:** Sergio Joaquín Álvarez-Méndez, Juan Luis Ramos-Suárez, Axel Ritter, Javier Mata González, Ángeles Camacho Pérez

**Affiliations:** a Departamento de Ingeniería Agraria y del Medio Natural. Universidad de La Laguna. La Laguna, Tenerife, Spain; b Instituto Universitario de Bio-Orgánica Antonio González, Universidad de La Laguna, Avda. Astrofísico Francisco Sánchez, 38206 La Laguna, Tenerife, Spain; c Área de Ingeniería Agroforestal, Universidad de La Laguna, Spain

**Keywords:** Biochemical methane potential, Bioplastics, Anaerobic digestion, PLA, PBAT

## Abstract

Bioplastics aim to substitute conventional plastics in most applications, a critical one being the collection of organic wastes for composting or anaerobic degradation. The anaerobic biodegradability of six commercial bags composed of PBAT or PLA/PBAT blends and certified as compostable [1] was studied using ^1^H NMR and ATR-FTIR techniques. This study aims to elucidate if commercial bioplastic bags are biodegradable under conventional conditions found in anaerobic digestates. Results showed that all studied bags are hardly anaerobically biodegradable at mesophilic temperatures. The biogas yield resulting from the anaerobic digestion under laboratory conditions oscillated between 270.3 ± 45.5 L kgVS^−1^ for a trash bag composed of 26.64 ± 0.03%/73.36 ± 0.03% PLA/PBAT and 36.7 ± 25.0 L kgVS^−1^ for a bag composed of 21.24 ± 0.08%/78.76 ± 0.08% PLA/PBAT. The degree of biodegradation did not correlate with PLA/PBAT molar composition. However, ^1^H NMR characterization showed that the anaerobic biodegradation occurred mostly in the PLA fraction. No bioplastics biodegradation products were detected in the digestate fraction (<2 mm). Finally, none of the biodegraded bags comply with the EN 13432 standard.

## Highlights

• ^1^H NMR showed the biodegradation of the PLA fraction in PLA/PBAT commercial bags.
• ^1^H NMR revealed no PLA or PBAT degradation by-products in digestate.
• PLA/PBAT ratios are not the main factor affecting anaerobic biodegradability.
• Mass loss was low for all commercial bags (1.79–24.61%).
• None of the bags comply the EN13432 standard for plastic anaerobic biodegradation.

## 1 Introduction

Plastic pollution is one of the most pressing problems today as a consequence of the high production of plastics and their inadequate management once used. Many plastics do not follow proper end-of-life management, representing pollution to the marine or terrestrial environments [2], where changes in their physicochemical characteristics are relevant to their environmental risk [3]. A large part of plastic pollution in soils can originate when using soil amendments, such as sewage sludge, compost or digestate, which may contain plastics and remain in the soil as macro- (>5 mm), micro- (<5 mm) or nanoplastics (<1 μm) [4]. In fact, regardless of the prevention methods applied at the waste management plants, microplastics were still found in digestates and composts produced from biowastes [5]. Therefore, it is urgently necessary to replace conventional plastics with bioplastics for Organic Fraction of Municipal Solid Waste (OFMSW) collection and food packaging.

**Fig. 1:**
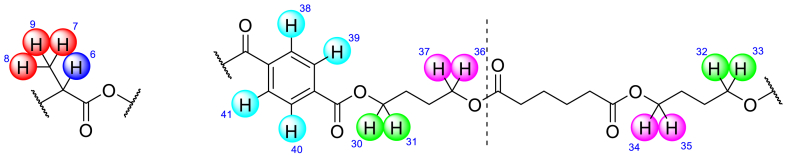
Monomer of PLA (left) and the two constituent monomers of the random copolymer PBAT (right). Key atoms for the ^1^H NMR identification are numbered and coloured (chemically equivalent protons are indicated with the same colour).

While the term bioplastics typically includes bio-based and biodegradable plastics, non-bio-based plastics can also be biodegradable. This is the case of poly (butylene adipate-*co*-terephthalate) (PBAT, Fig. 1), which is produced from petrochemicals [6]. On the other hand, bio-based plastics are typically biodegradable. These are of plant origin (e.g., starch), polymerized bio-monomers (e.g., polylactic acid -PLA-, Fig. 1) or extracted biopolymers (e.g., polyhydroxyalkanoates -PHA-) [7]. In 2021 1553 Mt of biodegradable plastics were produced, from which 29.9% and 29.4% corresponded to PBAT and PLA, respectively [8]. Both components are likely to dominate the market in the coming years, with the combination of both being a common methodology to produce high quality, biodegradable materials [7]. PBAT is a co-polyester made of butanediol (BDO), adipic acid (AA) and terephthalic acid (PAT), specifically made for increasing the hydrolytic susceptibility and biological degradability by introducing aliphatic components into the aromatic polyester chains [9]. PLA is a poly-α-hydroxy acid, a type of linear aliphatic thermoplastic polyester which can be produced either biologically or chemically with the help of bacteria [10]. The blend of PLA and PBAT is normally used to improve PLA properties. During blending, no chemical reactions take place [11]. However, PLA and PBAT are immiscible and therefore different strategies are used to increase their compatibility, such as chain extenders, additives or transesterification reactions [12]. The biodegradation of plastics depends on environmental conditions (temperature, moisture …) and the physical and chemical properties of the biopolymer (composition, molecular weight, crystallinity, chemical structure, hydrophilicity …) [13,7]. PLA biodegradation takes place through chemical hydrolysis at high humidity and at elevated temperatures, and the resulting oligomers and monomers can be metabolized by microorganisms [14]. Moreover, the rate of biodegradation of PLA depends on its chemical structure. There are three enantiomeric forms of PLA: levorotatory (L-), dextrorotatory (D-), and meso (a combination of L- and D-). A higher D-content decreases the rate of crystallization, which has been shown to be an important parameter for increasing the rate of biodegradation of PLA [10]. On the other hand, PBAT biodegradation rate depends on the amount of PTA in the polymer: higher PTA content decreases the biodegradation rate. With up to 50% PTA, the PBAT will degrade under composting [9]. Moreover, it has been shown that the oligomers and the monomers formed during biodegradation are completely metabolized without producing residues [14].

Biodegradation of plastics is expected to occur in any process normally used for organic wastes management. Specific standards have been developed for biodegradable plastics, such as [15,1]. However, they focus generally on the composting process, while anaerobic digestion is hardly considered. On the other hand, other standards cover the anaerobic biodegradability of plastics [16,17,18] and [19]. Nevertheless, these standards do not reproduce conditions that are normally found at industrial biogas plants [20,21].

According to previous studies the anaerobic biodegradability of different bioplastics is highly variable, even for bioplastics with the same main component [22,23,24]. Moreover, although the bioplastics comply with the EN 13432 standard, they might not biodegrade significantly under anaerobic conditions [21]. Only a few papers have already studied the biodegradation of the main marketable bioplastics blends of PLA and PBAT [25,26,27], whereas only Ref. [26] used commercial bioplastic bags. Ref. [27] found that PLA and PBAT films were hardly biodegradable under anaerobic conditions at a mesophilic temperature of 35 °C, with only 8.6% and 5.9% biodegradation, respectively. Ref. [25] showed similar results, with a mineralization rate of 4.6% and 2% for PLA and PBAT grinded pellets, respectively, under anaerobic conditions at a mesophilic temperature of 36 °C. Ref. [26] also showed the low biodegradation of a commercial bag made of PLA and PBAT under anaerobic conditions at mesophilic (35 °C) and thermophilic conditions (55 °C), with no biogas production.

The attenuated total reflectance coupled with Fourier-transform infrared (ATR-FTIR) technique is the most demanded spectroscopic analysis method for monitoring the bioplastic degradation process [28]. The solution-state nuclear magnetic resonance (NMR) is also an extremely useful spectroscopic tool which explores the magnetic properties of certain atomic nuclei (^1^H and ^13^C, mainly). ^1^H NMR has also been employed for evaluating the decomposition of PLA, PBAT and PLA/PBAT blends under various conditions: abiotic incubation in hot water [29,30,31] or paraffin [31]; burial in soil [32]; industrial composting [29]; and laboratory aerobic [33,34] and anaerobic procedures [34].

This study aims at evaluating the anaerobic biodegradability of commercial bioplastic bags composed of PBAT and PBAT/PLA blends. Commercial bioplastic bags were subjected to anaerobic digestion batch trials, where biogas production and biodegradability were evaluated. Finally, ^1^H NMR and ATR-FTIR were used to assess the way in which these bioplastics bags are biodegraded under anaerobic conditions.

## 2 Materials and methods

### 2.1 Substrates and inoculum

Six bioplastic bags were obtained from different public stores in Tenerife and Gran Canaria (Canary Islands, Spain). Five from these bags were collected at supermarkets: two being sold as specific trash bags (TB1; TB2), two were used as bags for fruit collection and weighing (FB1, FB2) and the other one was purchased as grocery bag (GB). The remaining bag was purchased from a pharmaceutical cooperative which delivers this type of bags to different pharmacies (PB). All bags were analysed in terms of total and volatile solids (TS, VS) and their composition was investigated by means of the ^1^H NMR and ATR-FTIR techniques, since no response was obtained from the manufacturers/dealers regarding their original composition. Moreover, no information on the manufacturing process was obtained.

The inoculum used for the anaerobic digestion was originally obtained from a full-scale, mesophilic anaerobic digester that treats sewage sludge from municipal wastewater. This inoculum has changed over two years as it has been used in other anaerobic assays treating cheese whey, pig manure and other organic wastes. Composition of the inoculum for the three different Biochemical Methane Potential (BMP) assays is shown in Table 1.

**Table 1:** Composition of the inoculum used throughout the three series of BMP assays. TS = total solids; VS = volatile solids; CODt = total chemical oxygen demand; CODs = soluble chemical oxygen demand; PA = partial alkalinity; TA = total alkalinity; A1 = Assay 1; A2 = Assay 2; A3 = Assay 3. Values are expressed as means±standard errors (n = 3).

	pH	TS (%)	VS (%)	CODt (gO_2_ L^−1^)	CODs (gO_2_ L^−1^)	PA (gCaCO_3_ L^−1^)	TA (gCaCO_3_ L^−1^)
A1	8.0 ± 0.0	5.5 ± 0.2	3.3 ± 0.2	66.4 ± 3.5	27.4 ± 0.5	13.0 ± 0.4	18.7 ± 0.5
A2	8.2 ± 0.0	3.7 ± 0.1	2.0 ± 0.1	34.6 ± 1.1	12.7 ± 1.4	14.3 ± 0.1	15.1 ± 0.1
A3	8.2 ± 0.0	2.7 ± 0.0	1.4 ± 0.0	21.9 ± 0.8	6.8 ± 0.2	8.7 ± 0.1	10.8 ± 0.2

### 2.2 Biochemical methane potential assays

Biochemical Methane Potential (BMP) assays were done in three series for the different bioplastic bags in a Biogas Batch Fermentation System (Dr.-Ing. RITTER Apparatebau GmbH & Co. KG, Germany). BMP assays were performed following the German Standard VDI 4630 (Verein Deutscher Ingenieure) [35] for the fermentation of organic material. Each substrate was mixed with inoculum in each reactor (1 L total capacity, 0.8 L working volume) preserving the ratio VS_substrate_/VS_inoculum_ < 0.5, according to the description shown in Table 2. All bags were manually cut with scissors in 2 × 2 cm pieces before introducing them into the reactors. Then, reactors were purged with nitrogen, closed, sealed, and left in anaerobic conditions at controlled temperature (37 °C) with constant agitation until assays finished at 50–70 days (i.e., when daily biogas production during more than 3 days was lower than 1% of the accumulated biogas production). Bag pieces were also added to an empty reactor to evaluate if changes occur without inoculum under the evaluated conditions. The gas produced in each reactor was collected in biogas bags and its composition was analysed periodically with a commercial biogas analyser (Multitec 545, Sewerin, Germany) which has infrared sensors for measuring methane (0–100%) and carbon dioxide (0–100%), and electrochemical sensors to measure hydrogen sulphide (0–5000 ppm) and oxygen (0–25%). Gas (biogas or methane) yield was calculated as follows:

$$Y_{gas}=\frac{P_{gas\;substrate}-P_{gas\;inoculum}}{{OM}_{substrate}}$$

where *Y*_*gas*_ (L kgVS^−1^) represents the gas yield; *OM*_*substrate*_ (kg VS) is the organic matter of the substrate introduced into the reactor; *P*_*gas substrate*_ (L) is the total volume of gas generated in each reactor; and *P*_*gas inoculum*_ (L) is the volume of gas produced by the inoculum in test reactors, which was calculated as the product of organic matter from the inoculum in each of the test reactors (in kg VS) and the average gas yield of the inoculum (in L kgVS^−1^) obtained in blank reactors, i.e., the ratio of the gas volume production in the blank reactor and its VS content.

**Table 2:** Details of the composition of each reactor in the three series of Biochemical Methane Potential assays performed to evaluate biodegradation and biogas production from each bioplastic bags. A = Assay; R = Reactor; GB = Grocery bag; TB = Trash bag; PB=Pharmacy bag; FB=Fruit collection and weighting bag.

	Bioplastic Bag	Inoculum (g)	Inoculum (gVS)	Substrate (g)	Substrate (gTS)	Substrate (gVS)
A1-R1 & R2	GB	800	26.29	22.61	22.57	13.15
A1-R3 & R4	TB1	800	26.29	13.58	13.27	13.15
A2-R1 & R2	PB	800	15.78	10.14	10.10	7.89
A2-R3 & R4	FB1	800	15.78	8.76	8.58	7.89
A3-R1 & R2	FB2	800	11.12	6.10	6.00	5.56
A3-R3 & R4	TB2	800	11.12	5.73	5.61	5.56

Once BMP assays finished, the content of the reactors was filtered through a 2 mm mesh size sieve to retain the bioplastic pieces and to recover the inoculum. Inoculum was analysed before and after the assays to determine physical and chemical parameters according to section 2.3.4. Bioplastics were cleaned (first, with tap water and, afterwards, with distilled water) and air dried for 7 days. Since a lot of bioplastic pieces entered each reactor, this operation was performed with a representative number of bioplastic pieces from each reactor. Cleaned and dried bioplastic bags were weighted to quantify weight loss according to the final and initial masses. These results were extrapolated to all bioplastic pieces added to each reactor. Afterwards, they were analysed in ^1^H NMR and ATR-FTIR to study compositional changes as described below. All bioplastic bags were analysed in duplicates (see Fig. S1 for step-by-step pictures of the process).

### 2.3 Analytical methods

#### 2.3.1 Chemical drawings

Molecules were drawn and atom-numbered using PerkinElmer ChemDraw® Professional version 19.1.0.8.

#### 2.3.2 Stereochemical analysis

PBAT and PBAT/PLA blends were insoluble in *n*-hexane, ethyl acetate, diethyl ether, acetone, methanol, ethanol, acetic acid and acetonitrile. They were successfully solved in dichloromethane or chloroform (CHCl_3_), although high dilutions were required to avoid milky solutions unable to be analysed in the polarimeter. Thus, 1 mg of each bioplastic was solved in 10 mL of CHCl_3_ and optical rotations were recorded at 25 °C using a PerkinElmer 343 polarimeter with a sodium lamp operating at 589 nm.

#### 2.3.3 ^1^H NMR analysis

Samples were prepared in triplicate according to the following procedure: a) 5 mL of the aqueous inoculum obtained after BMP assays was poured into a separatory funnel together with 5 mL of brine and 40 mL of CHCl_3_. After vigorous shaking, the lower organic layer was separated and the upper aqueous layer was successively extracted with two new portions of 40 mL of CHCl_3_. The combined organic layers were washed with 50 mL of brine, dried over anhydrous MgSO_4_, filtered, concentrated in a rotary evaporator and finally dried under high vacuum. Alternatively, 5 mL of the aqueous inoculum was lyophilized (ALPHA-2-4, Martin Christ Gefriertrocknungsanlagen GmbH, Germany) and used without further purification. In both cases, the solid crude was solved into 0.7 mL of CDCl_3_, an isotopically enriched form of CHCl_3_, and analysed as described below. b) 5 mg of each studied bioplastic (both prior to BMP assays and after them) was solved into 0.7 mL of CDCl_3_ and analysed as described below.

Samples analysis: ^1^H NMR (500 MHz) spectra were recorded on a Bruker Advance instrument at room temperature, and data were processed using Topspin software version 4.0.9.^1^H NMR spectra are referenced to the resonance from residual CHCl_3_ at 7.26 ppm. Chemical shifts (*δ*) are given in parts per million (ppm), and coupling constants (*J*) are expressed in hertz. Multiplicity is expressed by the abbreviations m (multiplet), br (broad signal), d (doublet) and q (quartet). Structure elucidation was made using the two-dimensional NMR techniques correlation spectroscopy (COSY), edited heteronuclear single-quantum correlation spectroscopy (HSQC) and heteronuclear multiple-bond correlation spectroscopy (HMBC) (see Fig. S2).

**Fig. 2:**
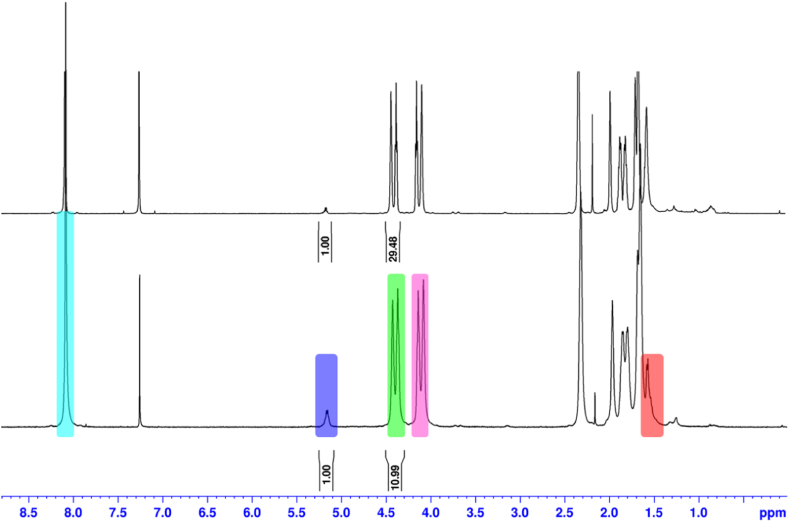
Example of ^1^H NMR monitoring of the degradation of a PLA/PBAT blend: initial (down) and final (up) ^1^H NMR spectra of commercial trash bag (TB1), with coloured key signals of constituent biopolymers (see corresponding-coloured protons in Fig. 1).

Biopolymers characterization: PBAT ^1^H NMR (500 MHz, *δ*, CDCl_3_): 1.66–1.69 (m, 6H), 1.80–1.86 (m, 4H), 1.97 (br s, 2H), 2.32 (br s, 4H), 4.08–4.14 (m, 4H), 4.37–4.43 (m, 4H), 8.09 (m, 4H) ppm; PLA ^1^H NMR (500 MHz, *δ*, CDCl_3_): 1.58 (d, *J* = 7.0 Hz, 3H), 5.16 (q, *J* = 7.0 Hz, 1H) ppm. The comparison of the integrals associated to key signals (*δ* = 4.37–4.43 ppm for H30–H33 of PBAT and *δ* = 5.16 ppm for H6 of PLA, see Fig. 1, Fig. 2) of the constituent biopolymers allowed for obtaining their ratio. Relative variation of PLA (%) in PLA/PBAT blends after and before BMP assays was calculated according to the final and initial PLA molar masses (expressed in %).

#### 2.3.4 ATR-FTIR analysis

A PIKE MIRacle™ Single Reflection ATR sampling accessory equipped with a diamond crystal was used for near IR spectral analysis. After 20 background scans, 20 sample scans were carried out with a spectral resolution of 4 cm^−1^ within a range of 4000 to 650 cm^−1^. Key signals for each biopolymer were expressed in cm^−1^.

#### 2.3.5 Substrate and inoculum physical-chemical composition

TS and VS were analysed according to Ref. [36]. Partial and total alkalinity (PA, TA) were determined according to Ref. [37] by titration to pH 5.75 and 4.3, respectively. Intermediate alkalinity (IA) was calculated as the difference between TA and PA. Total and soluble chemical oxygen demand (COD_t_, COD_s_) were analysed by an adaptation of the 410.4 method of U.S. EPA, using a multiparametric photometer HI83399 (Hanna Instruments; Woonsocket, USA). Analyses were performed in triplicates per sample.

### 2.4 Statistical analysis

The Mann–Whitney *U* test was used with the software SPSS Statistics version 26.0.0.0 (IBM Corporation, Armonk, New York, USA) to assess significant differences.

## 3 Results and discussion

### 3.1 Substrate characterization

All the studied bioplastic bags were certified as EN 13432, and their flexibility and resistance seemed very different at first sight, which shows the importance of the manufacturing process in the bioplastic properties. The ^1^H NMR analysis showed that all of them were made of PLA and PBAT in different proportions, except for the grocery bag (GB), which was made entirely from PBAT (Table 3). Thus, except for GB, all bags are likely made of ecovio® by BASF, which according to their website can be obtained from different proportions of PBAT and PLA depending on the final use (organic waste collection, carrier bags, fruit and vegetable bags, among others). Optical rotations were measured for all the studied bags and no optical activity was detected before nor after the anaerobic digestion process, thus revealing that PLA was in a racemic form. GB was the bag with the lowest VS content (<60%). A low VS content (<80%) was also observed in PB, which was composed of both biopolymers at approximately 50%. The rest of the bioplastic bags presented VS content higher than 90% (Table 3). All of them, despite the low VS content of GB, comply with the requirements of EN 13432 regarding the VS content of biodegradable polymers, which should be above 50%VS content.

**Table 3:** Initial solids content and initial and final molar composition (%) of the six bioplastic bags.

		Initial composition				Final composition after digestion		Variation	
Bag type	DA (days)	TS (%)	VS (%)	PBAT (%)	PLA (%)	PBAT (%)	PLA (%)	PLA loss (%)	Mass loss (%)
TB1	70	97.74 ± 0.05	96.84 ± 0.06	73.36 ± 0.03	26.64 ± 0.03	88.23 ± 0.13	11.77 ± 0.13	55.82*	24.61**
GB	70	99.82 ± 0.00	58.15 ± 0.17	100.00 ± 0.00	0.00 ± 0.00	100.00 ± 0.00	0.00 ± 0.00	0.00	1.79**
TB2	63	97.97 ± 0.03	97.04 ± 0.40	65.52 ± 0.00	34.48 ± 0.00	74.66 ± 0.09	25.34 ± 0.09	26.51*	14.18**
FB1	52	97.92 ± 0.18	90.11 ± 0.81	86.68 ± 0.64	13.32 ± 0.64	91.90 ± 0.08	8.10 ± 0.08	39.16**	5.67**
FB2	63	98.36 ± 0.01	91.20 ± 0.01	78.76 ± 0.08	21.24 ± 0.08	85.23 ± 0.18	14.77 ± 0.18	30.45*	3.44**
PB	52	99.67 ± 0.05	77.85 ± 3.16	52.46 ± 0.64	47.54 ± 0.64	63.22 ± 0.28	36.78 ± 0.28	22.62**	3.40**

DA: duration of BMP assay (days); PBAT: poly(butylene adipate-*co*-terephthalate); PLA: polylactic acid; TS: total solids; VS: volatile solids. Values are expressed as means±standard errors (n = 6); asterisks indicate statistically significant differences at the 0.05 (*) or 0.01 levels (**) between final and initial mass or PLA content according to Mann–Whitney *U* test.

### 3.2 ^1^H NMR characterization

Fig. 2 shows the spectra of a piece of TB1 prior (down) and after (up) a BMP assay. Both spectra were very clean and exhibited an excellent signal-to-noise ratio, with no background noise hindering the identification and integration of the key signals. The PLA to PBAT ratios were established according to the integral ratios of signals at *δ* = 4.37–4.43 ppm (this area must be divided by 4 because it corresponds to the equivalent protons H30–H33 of PBAT, which are green marked in Figs. 1) and 5.16 ppm (corresponding to the proton H6 of PLA, marked in dark blue in Fig. 1). Thus, commercial TB1 was originally made of a 1/2.75 mixture of PLA/PBAT, i.e., 26.64/73.36 of molar proportion. After the anaerobic digestion process, the bioplastic TB1 exhibited a notable decrease in the intensity of PLA signals, yielding a 7.37/1 ratio (i.e., 88.23/11.77 M proportion). Therefore, in TB1 the PLA degradation was faster than that of PBAT degradation under the studied conditions.

Table 3 showed the final molar composition (in percentage for PBAT and PLA) after the BMP assays for each of the bags analysed. After biodegradation, all bioplastic bags made from PBAT and PLA showed a decrease in the molar percentage of PLA and a consequent increase in the molar percentage of PBAT. TB1 had the highest reduction in PLA (55.82%), followed by FB1 and FB2 (39.16 and 30.45%, respectively). On the other hand, PB exhibited the lowest relative decrease in PLA (only 22.62%). Results clearly showed that in commercial bags made from a mixture of PBAT and PLA, the anaerobic biodegradation of PLA was higher than that of the PBAT. None of the studied bags revealed molecular changes by ^1^H NMR when they were heated in the same conditions of the BMP assays but without adding inoculum, revealing that the observed decrease of PLA was not a mere consequence of the prolonged heating.

According to the scientific literature, PLA or PBAT content changes after biodegradation strongly depend on the studied conditions. On the one hand [34], found no differences in PLA and PBAT polymers (2 × 2 cm films) when they were separately degraded under liquid phase aerobic and anaerobic standard ISO tests, on the basis of ATR-FTIR and ^1^H NMR spectroscopy. Similarly, Ref. [32] obtained for PLA films (20 × 25 cm) identical ATR-FTIR and ^1^H NMR spectra before and after being buried at a 5 cm depth in soil during a one-year period. According to Ref. [26], a commercial bag made from PBAT and PLA showed no anaerobic biodegradation and the ATR-FTIR spectra remained unchanged after 100 days in a batch anaerobic biodegradation assay under mesophilic temperature. On the other hand, several authors observed changes in the PLA/PBAT ratio after a degradation process. For instance, Ref. [33] found a degradation rate of 18.95 w_t_% in PLA/PBAT blends (20/80 w/w, 1 × 1 cm films) exposed for 5 days to a co-culture of *Pseudomonas mendocina* and *Actinomucor elegans* in an enriched medium shaken and maintained at 30 °C. These authors did not report if the PLA/PBAT ratio in the mixture changed during the degradation process, albeit ATR-FTIR and ^1^H NMR revealed the apparition of olefinic signals (at 1635 cm^−1^ and 4.88 ppm, respectively), as consequence of a degradation mechanism which involves proteases- and lipases-mediated cleavage of the ester bonds of PLA and PBAT, respectively. Ref. [27] studied the degradation of PLA, PBAT and their blends (<1 cm), and also observed a higher degradation for PLA, which was also noticed in ATR-FTIR spectra. However, no determination of biodegradation of the different components in the mixture could be determined. Ref. [29] analysed by ^1^H NMR two kinds of PLA/PBAT blends (a 17/83 mol% film and a 40/60 mol% disposable market bags, with 0.02 and 0.1 mm thickness, respectively) before and after an abiotic incubation (70 °C), achieving total degradation of PLA after 42 and 70 days, respectively. Moreover, they also remarked the importance of the material preparation procedure and the degradation environment, since a higher PLA degradation rate was found in a Kneer container composter system than in hot water. Similarly, Ref. [31] incubated dumbbells of PLA/PBAT (12/88 mol%, 1.5 mm thickness) in demineralized water at 70 °C for 84 days finding complete degradation of PLA, and also the appearance of smaller by-products as consequence of the selective cleavage of the ester bonds of the PBAT aliphatic part. Ref. [30] (PLA/PBAT 25/75 mol%, pieces of 0.7 g, hot water incubation at 70 °C during 70 days) reached the same conclusions.

Our study showed that biodegradation can occur at mesophilic temperatures for different blends of PBAT and PLA commercial bags, and that this biodegradation occurs mostly in the PLA fraction under the studied conditions. Thus, these results are in line with Refs. [27,31]; and Refs. [29,30]. Moreover, Refs. [30,31]; and also Ref. [33] reported the appearance of degradation by-products in the PLA/PBAT blends based on the ^1^H NMR analysis. Contrary to these studies, no by-products were detected under our experiment conditions (Fig. 2). This is probably related to the fact that they used microorganisms that specifically degrade PLA/PBAT [33] or that the experiments were performed at higher temperatures [30,31].

**Fig. 3:**
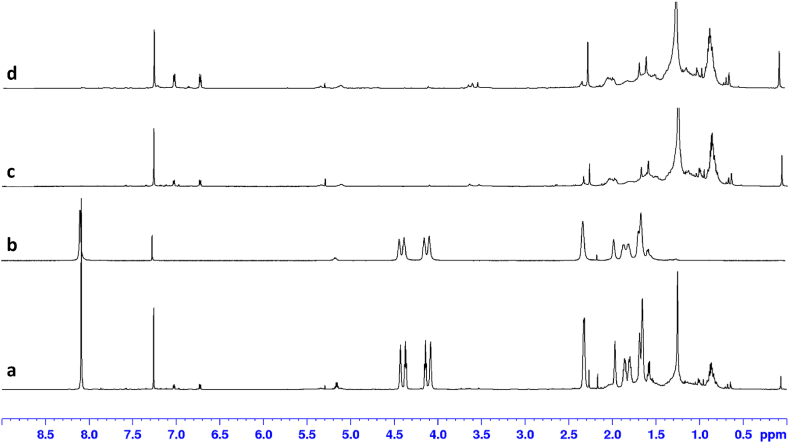
Evaluation of the absence of PBAT and PLA in the extracted aqueous phase after anaerobic digestion process: a) inoculum from a blank reactor after a BMP assay plus 5 mg of commercial trash bag (TB1) added to the CHCl_3_ used for its extraction (positive control); b) commercial bioplastic TB1 (reference); c) inoculum from a blank reactor after a BMP assay (negative control); d) extracted digestate obtained after anaerobic digestion (70 days) of bioplastic TB1 (target).

According to Ref. [5], after anaerobic degradation microplastics can remain in the digestate and cause soil contamination if it is applied to agricultural fields. In this context, Ref. [33] analysed the components of the aqueous system from the degradation of PLA/PBAT blends mediated by coculture of *P. mendocina* and *A. elegans*. Liquid chromatography–mass spectrometry (LC–MS) allowed them to detect lactic acid oligomers (from degradation of PLA mainly mediated by *P. mendocina*), as well as terephthalic acid monomers and butanediol oligomers (from decomposition of PBAT mainly mediated by *A. elegans*). In our research, an additional NMR study was performed to evaluate if PBAT and/or PLA ends up in the liquid fraction of digestates at the studied anaerobic conditions (Fig. 3). A fraction of a blank inoculum used as control in the BMP assays was extracted with an organic solvent containing a previously dissolved piece of TB1. The resultant mixture was analysed by ^1^H NMR yielding spectrum “a” (see “samples preparation” in section 2.3.2 for further details). Its spectrum is the sum of spectrum “b” (commercial bioplastic TB1 showing the characteristic signals of both PLA and PBAT components, therefore, bioplastic remains unaltered during the extraction protocol) and spectrum “c” (pure inoculum spectrum with signals mainly between *δ* = 0.6–2.4 ppm, i.e., in the region of aliphatic protons). By coincidence, inoculum showed a signal at *δ* = 5.10 ppm, very closed to the *δ* = 5.15 ppm signal of H6 from PLA, but with different multiplicity and undoubtedly different to it, as illustrated in the amplification of the region *δ* = 3–8 ppm (see Figure S3). The spectrum of the extracted digestate obtained after a BMP assay of TB1 (“d”) is practically identical to the spectrum of pure inoculum (“c”). Thus, as none of the inherent signals of PBAT or PLA were detected by the highly sensitive NMR technique, it can be claimed that digestate remained free of unaltered, dissolved, or broken in tiny pieces, biopolymers under the studied anaerobic conditions. In any case, the low biodegradation observed for the bioplastic pieces, regardless of its composition, suggests that a contamination of bioplastics can occur if no composting of the solid fraction of the digestate takes place before its application to soils.

### 3.3 ATR-FTIR spectra

Spectra of the studied bioplastic bags were recorded by ATR-FTIR before and after the BMP assays, obtaining in both cases similar main peaks at those reported by Ref. [26] for PLA/PBAT blends: 2920 (*C*–H stretching of alkyl chains), 1720 (C=O stretching of ester groups), 1410 (*C*–H bending of PBAT methylene groups), 1268, 1101 and 1017 (*C*–O stretching of ester groups), and 870 and 728 (C=C bending of the PBAT aromatic ring) cm^−1^. As bag GB was made only from PBAT, an identical spectrum to the initial was obtained after anaerobic digestion process, confirming the absence of degradation by-products revealed by ^1^H NMR. On the other hand, the only remarkable differences in PLA/PBAT bags spectra were the decrease of the signal at 1017 cm^−1^ and the slight increase of that at 1410 cm^−1^, probably as consequence of the impoverishment in PLA (see Figure S4).

### 3.4 Biochemical Methane Potential assay

**Fig. 4:**
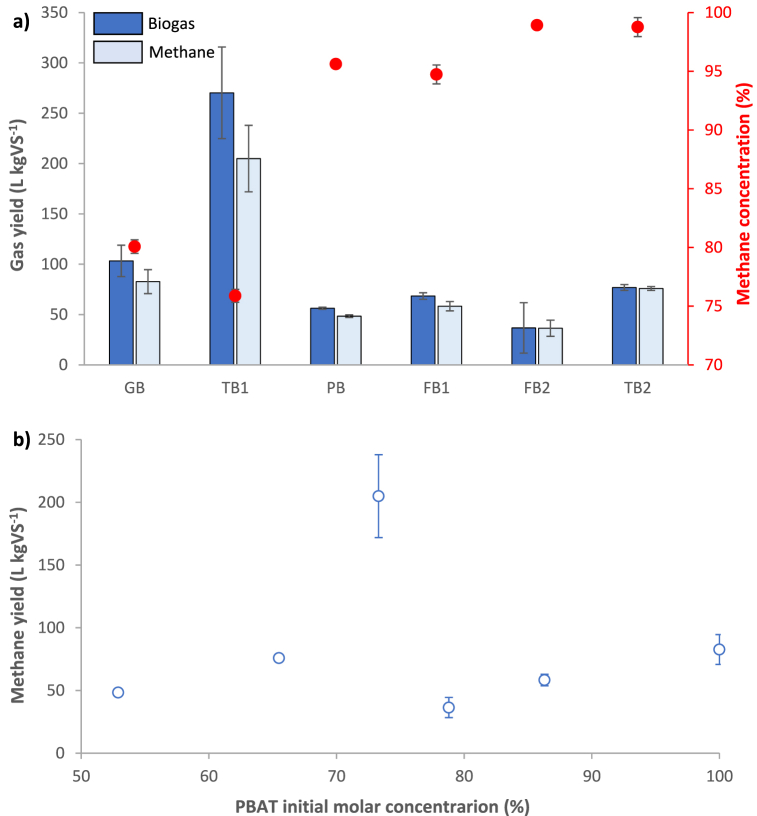
a) Average biogas and methane yields obtained with anaerobic digestion of the different bioplastic bags: GB (grocery bag), trash bag (TB1), PB (pharmacy bag); FB1 (fruit collection bag #1); FB2 (fruit selection bag #2); TB2 (trash bag #2). b) Average methane yield vs. composition of commercial bioplastic bags (initial molar concentration of PBAT expressed as percentage). Error bars represent the standard deviation. Biogas and methane production from blank reactors was subtracted.

BMP assay was performed in duplicate for each bioplastic bag. Biogas production was low for all bags (Fig. 4), except for TB1, whose degradation produced relatively high biogas and methane yields (270.3 ± 45.5 and 205.0 ± 33.0 L kgVS^−1^). The lowest biogas and methane yields were obtained for FB2 (36.7 ± 25.0 and 36.4 ± 8.0 L kgVS^−1^, respectively). GB, composed of 100% PBAT, had biogas and methane yields similar to the other commercial bags, although these are made from a blend of PBAT and PLA. Moreover, biogas and methane yield did not show any correlation with molar composition of PLA/PBAT (Fig. 4b). There are contradictory results regarding anaerobic biodegradability and biogas production from bioplastics in the scientific literature. Ref. [38] showed that PLA degrades completely in 60 days at 55 °C under anaerobic conditions, however, they did not observe degradation until day 55 at 35 °C. Ref. [39] also observed a higher PLA mineralization at higher temperatures, reaching a 60% mineralization after 40 days at 55 °C or after 100 days at 37 °C. Contrarily, Ref. [25] found only a 4.6% mineralization under anaerobic conditions at 36 °C after 80 days. Ref. [40] pointed out that PLA should be hydrolysed before microbial attack due to its high molecular weight, which can indicate why higher temperature favours anaerobic biodegradability of PLA. According to these variations, composition of the blend is not the only factor influencing anaerobic biodegradability of commercial bioplastic bags, rather physical properties, manufacturing process and the temperature at which anaerobic digestion takes place are probably similarly important. In fact, Ref. [34] already pointed out that the length of the polymer chain, the crystallinity degree and the complexity of the formula (e.g., presence of aliphatic rings) can influence biodegradability in a great extent.

At mesophilic temperature our study has shown that biogas can be obtained from biodegradation of PLA/PBAT mixtures and, according to ^1^H NMR analysis, most biogas came from PLA biodegradation. This is contradictory to Ref. [26] who did not observe any biogas production in PLA/PBAT mixtures but is in line with Ref. [27] who observed biogas production for PBAT and PLA as unique substrates under mesophilic anaerobic digestion assays. The fact that the degradation of the PBAT-based bag showed biogas production in our study is surprising, since according to reference institutions [41], PBAT is not biodegradable under anaerobic conditions. The biogas production from PBAT as a single substrate is contrary to what was observed in the biogas production from the PLA/PBAT mixture, which came mainly from the PLA fraction as indicated by the ^1^H NMR studies. It is possible that during PBAT monodigestion, the absence of another substrate available for microorganisms forced them to anaerobically degrade this component. However, this is only a hypothesis and other analyses, which are outside the scope of this study, would be required in order to draw conclusions from this result.

However, results also showed that none of the PLA/PBAT mixtures would pass the EN 13432 test, which specifies the criteria for the anaerobic biodegradation of plastics as a minimum of 50% degradation within a maximum of 2 months. Results of mass loss in bioplastic pieces at the end of the anaerobic assays (Table 3) are in accordance with biogas yields (i.e., higher biogas yields are associated with higher mass loss) for all bags except for GB (100%PBAT) which showed very low mass loss (1.8%) but significant biogas production (103.3 ± 15.6 L kgVS^−1^). In general, mass loss is low for all bioplastic bags, a sign of the low biodegradability of PBAT and PLA/PBAT commercial bioplastics under anaerobic conditions.

## 4 Conclusions

The anaerobic biodegradability study of commercial bioplastic bags using the ^1^H NMR and ATR-FTIR techniques was successful, and showed that the commercial bags had a different biopolymers composition (PLA/PBAT) that ranged between 0%/100% and 47.54 ± 0.64%/52.46 ± 0.64%, and that there were no degradation by-products after anaerobic digestion. Although PLA was more sensitive than PBAT to anaerobic biodegradation, none of the degraded bags met the EN 13432 standard for the anaerobic biodegradation of plastics. Since no correlation was found between PLA/PBAT ratio and biogas yields, it is concluded that the composition of the bag was not the only factor that affected anaerobic biodegradability.

*** E-supplementary data of this work can be found in online version of the paper.

## Author contribution statement

Sergio Álvarez-Méndez, PhD; Juan Luis Ramos Suárez, PhD: Conceived and designed the experiments; Performed the experiments; Analysed and interpreted the data; Wrote the paper.

Axel Ritter Rodríguez, PhD: Analysed and interpreted the data; Contributed reagents, materials, analysis tools or data; Wrote the paper.

Javier Mata González, PhD; Ángeles Camacho Pérez: Contributed reagents, materials, analysis tools or data; Wrote the paper.

## Data availability statement

Data included in article/supp. Material/referenced in article.

## Additional information

Supplementary content related to this article has been published online at [URL].

## Declaration of competing interest

The authors declare that they have no known competing financial interests or personal relationships that could have appeared to influence the work reported in this paper
