# Supplementary material for: Anaerobic digestion of commercial PLA and PBAT biodegradable plastic bags: Potential biogas production and 1H NMR and ATR-FTIR assessed biodegradation
Source: Heliyon. 2023 May 26;9(6):e16691. doi: 10.1016/j.heliyon.2023.e16691 (PMC10248121; doi:10.1016/j.heliyon.2023.e16691)
Supplement: Multimedia component 1 [file mmc1.docx]

**Supporting Information**

**Anaerobic digestion of commercial biodegradable bags: potential biogas production and ^1^H NMR-assessed biodegradation**

Sergio Joaquín Álvarez-Méndez^a,b^, Juan Luis Ramos-Suárez*^a^, Axel Ritter^c^, Javier Mata González^a^, Ángeles Camacho Pérez^a^

^a^ Departamento de Ingeniería Agraria y del Medio Natural. Universidad de La Laguna. La Laguna, Tenerife, Spain

^b^ Instituto Universitario de Bio-Orgánica Antonio González, Universidad de La Laguna, Avda. Astrofísico Francisco Sánchez, 38206 La Laguna, Tenerife, Spain

^c^ Área de Ingeniería Agroforestal, Universidad de La Laguna, Spain

* Corresponding Author:

Email: [jramossu@ull.edu.es](mailto:jramossu@ull.edu.es)

Full postal address: Sección de Ingeniería Agraria de la EPSI ULL. Cam. San Miguel de Geneto, 2, 38296 San Cristóbal de La Laguna, Santa Cruz de Tenerife

**Index**

[Figure S1. Pictures of the experimental protocol 3](#_Toc124186935)

[Figure S2. ^13^C NMR spectrum and two-dimensional NMR spectra of the PBAT-made commercial bag 5](#_Toc124186936)

[Figure S3. Ampliated region (δ = 3-8 ppm) of ^1^H NMR spectra shown in Figure 3 7](#_Toc124186937)

[Figure S4. Selected ATR-FTIR spectra 8](#_Toc124186938)

[Figure S5. Selected ^1^H NMR spectra of the six studied bioplastic bags before and after the anaerobic digestion 9](#_Toc124186939)

# Figure S1. Pictures of the experimental protocol


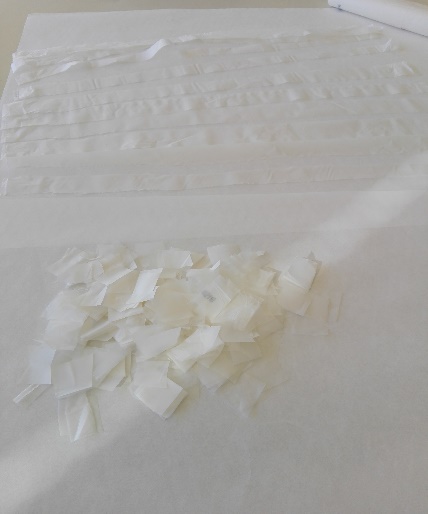


**a**


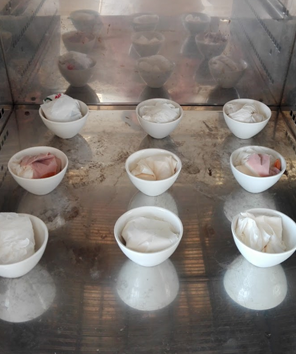


**b**


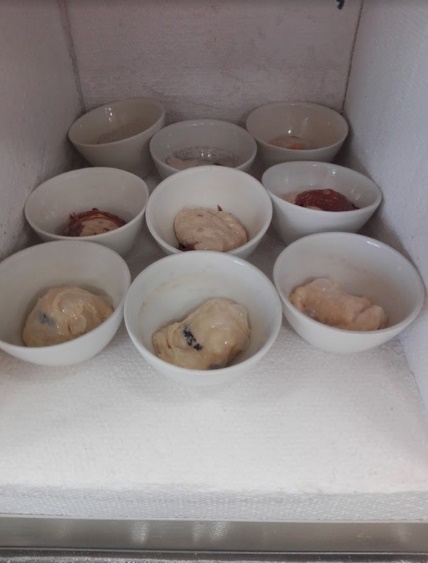


**c**


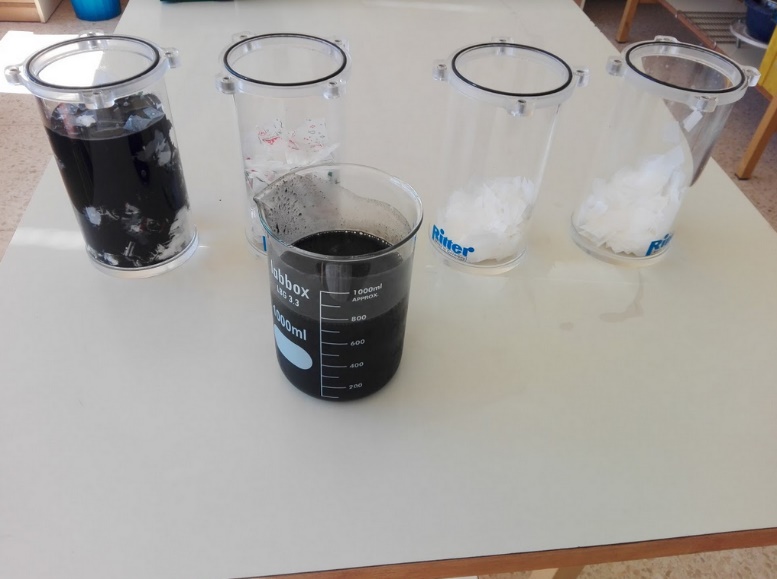


**d**


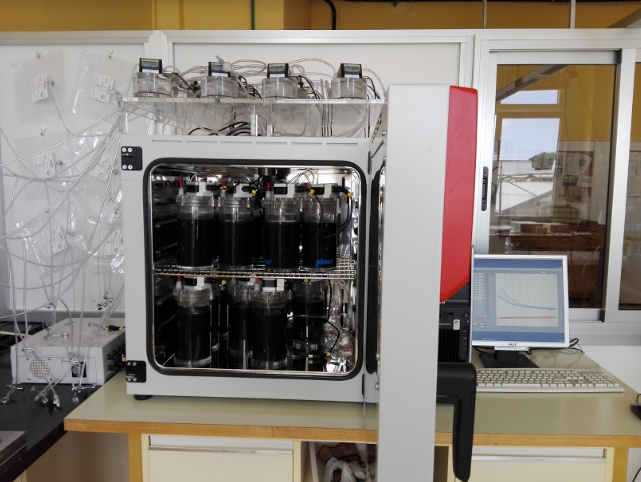


**e**


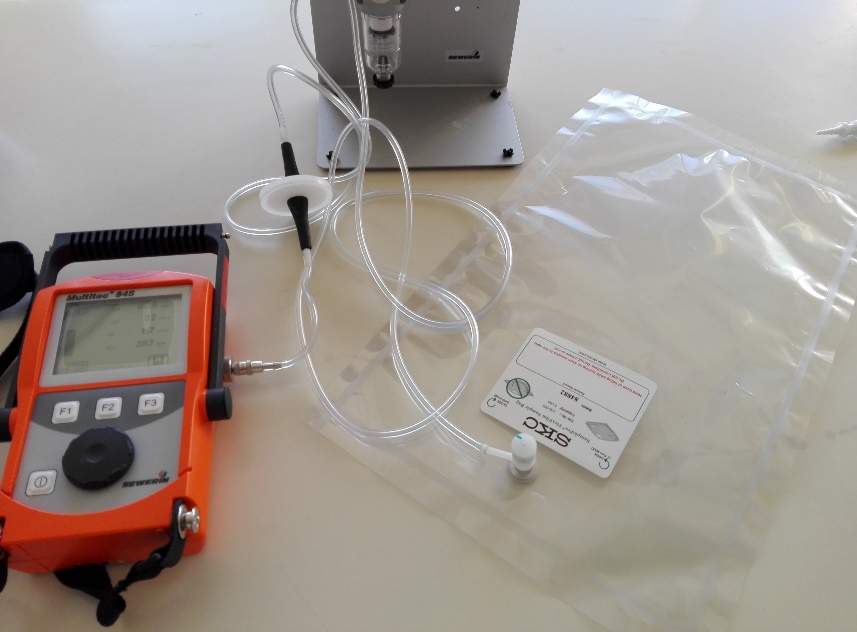


**f**


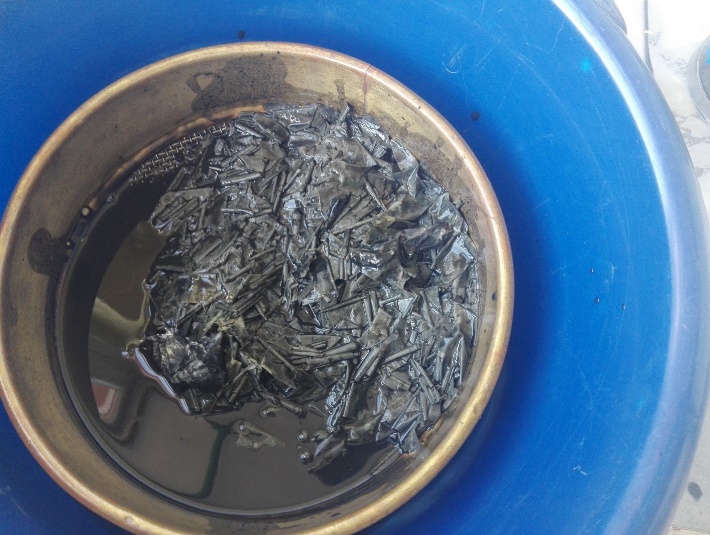


**g**

a) Cutting the commercial bags; b) heating the samples in an oven to calculate total solids (TS); c) incinerating the samples in a muffle to calculate volatile solids (VS); d) mixing the samples with the anaerobic inoculum preserving the ratio VSsample/VSinoculum < 0.5; e) running the biochemical methane potential (BMP) assay; f) measuring the biogas composition; g) filtering the samples once BMP assay finished.

**Figure S1** (continuation). Pictures of the experimental protocol


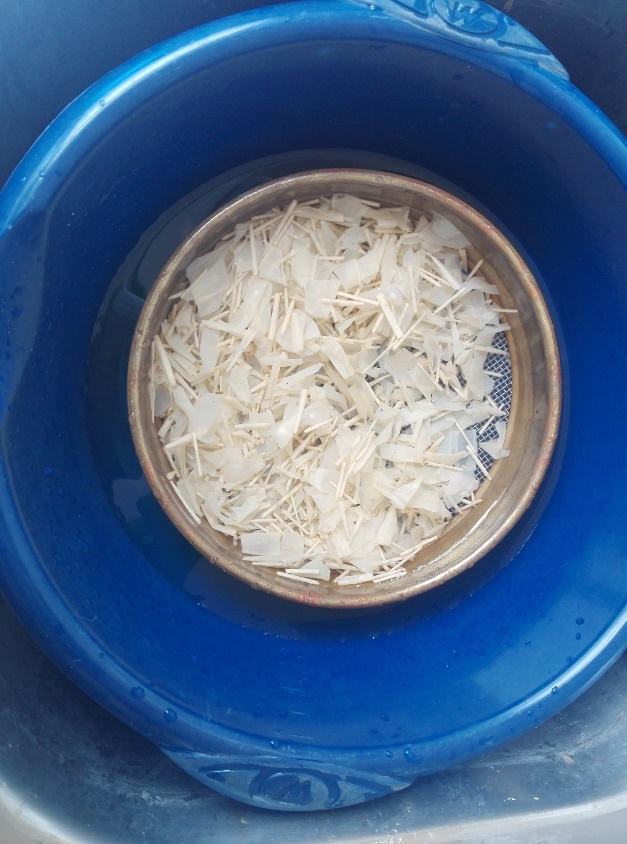


**h**


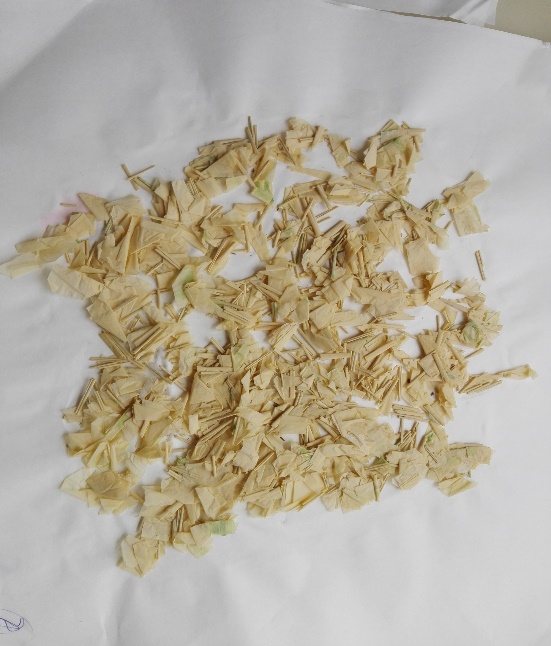


**i**


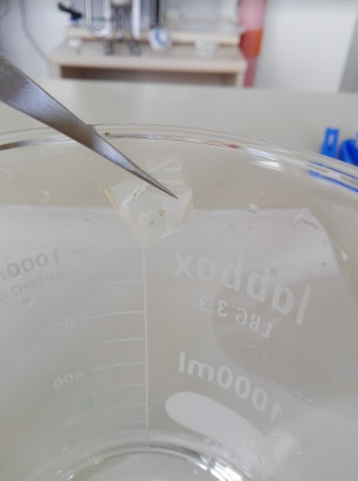


**j**


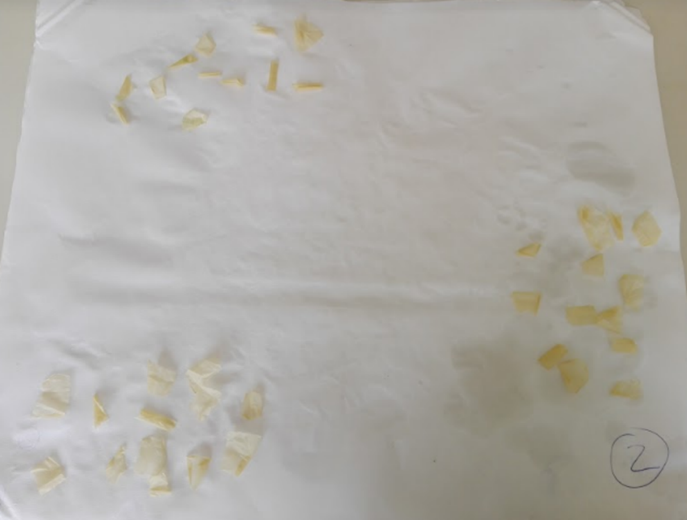


**k**


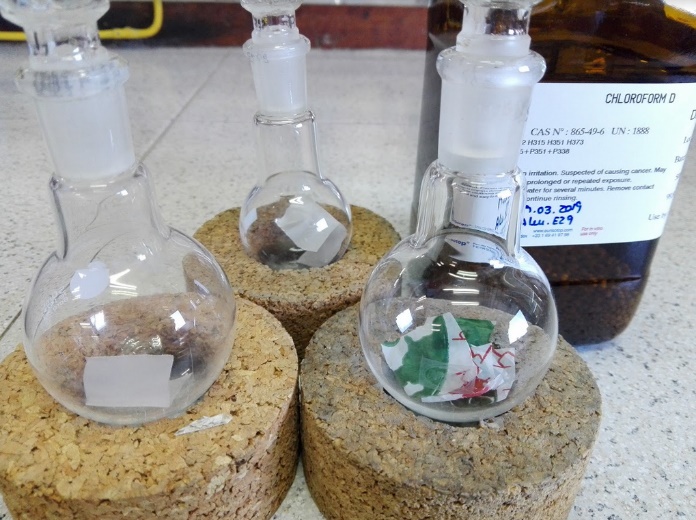


**l**


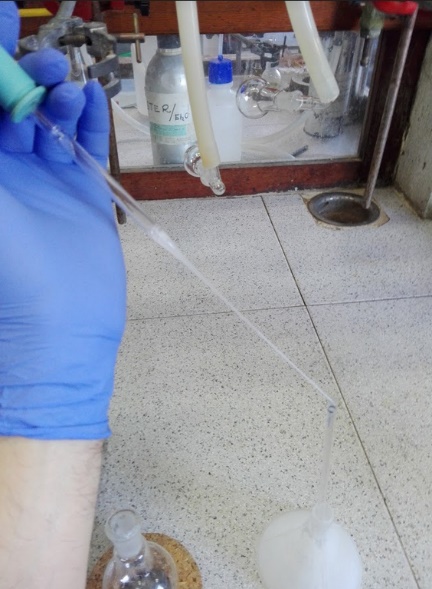


**m**

h) Prewashing the samples; i) air-drying the samples until constant weight; j) thoroughly washing a 0.2 g aliquot of the sample; k) air-drying the aliquots until constant weight; l) solving the clean samples in CDCl_3_; m) introducing the solved sample into a tube for nuclear magnetic resonance (NMR) analysis.

# Figure S2. ^13^C NMR spectrum and two-dimensional NMR spectra of the PBAT-made commercial bag

**a**


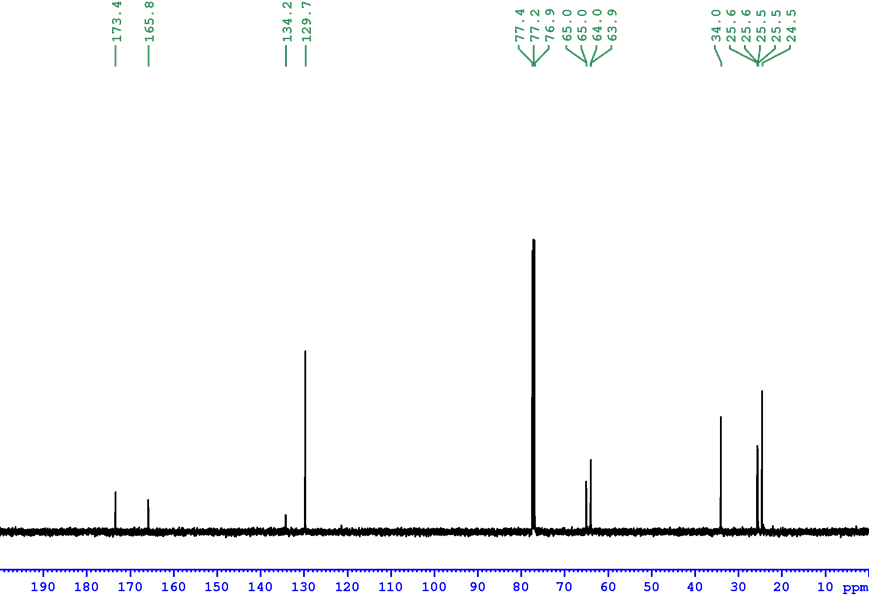

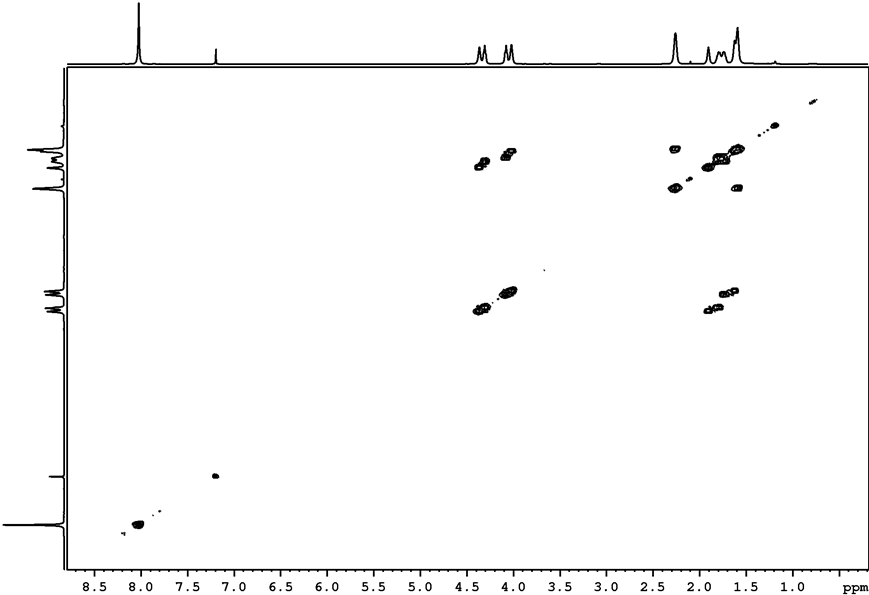


**b**

**c**


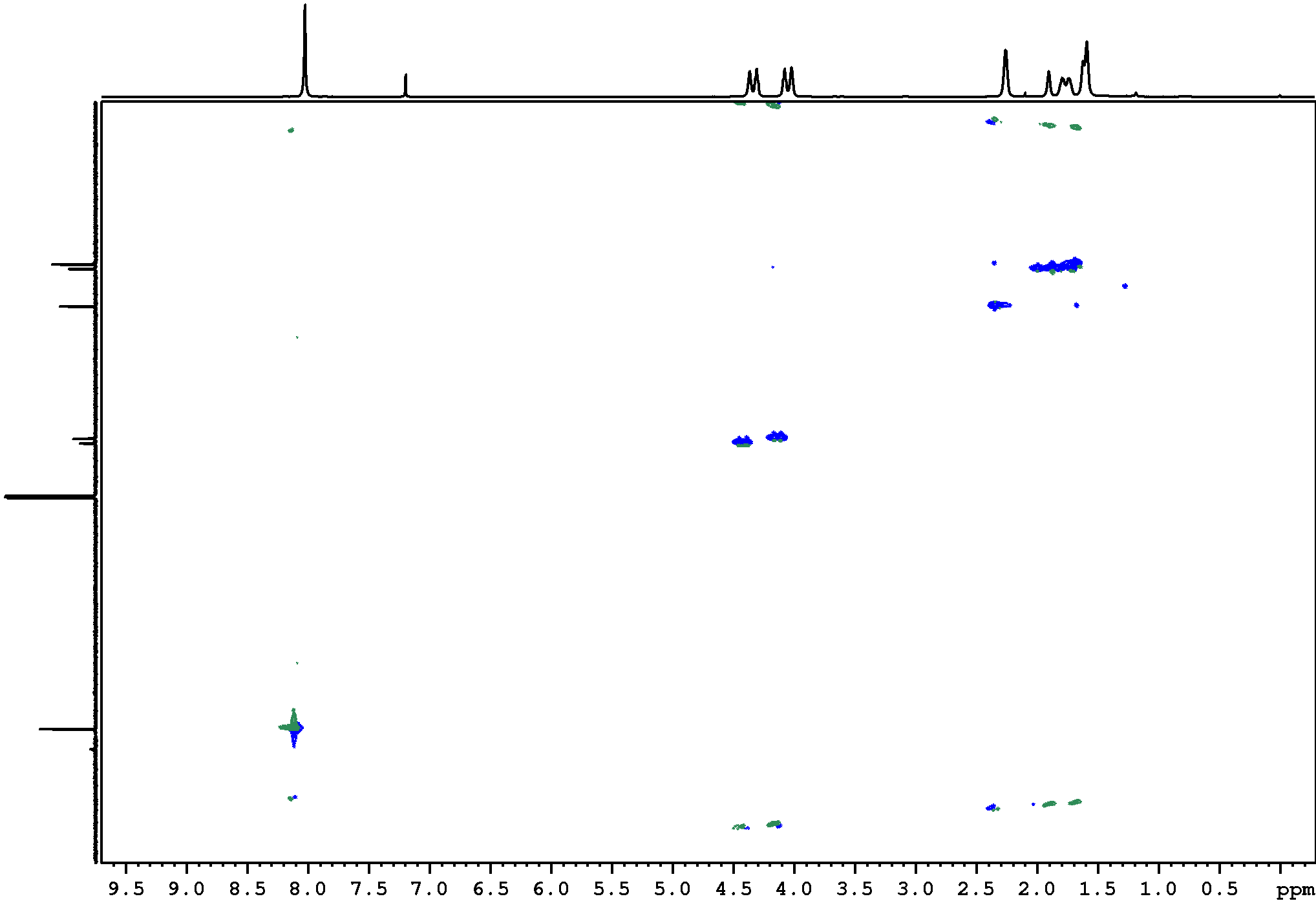


**d**


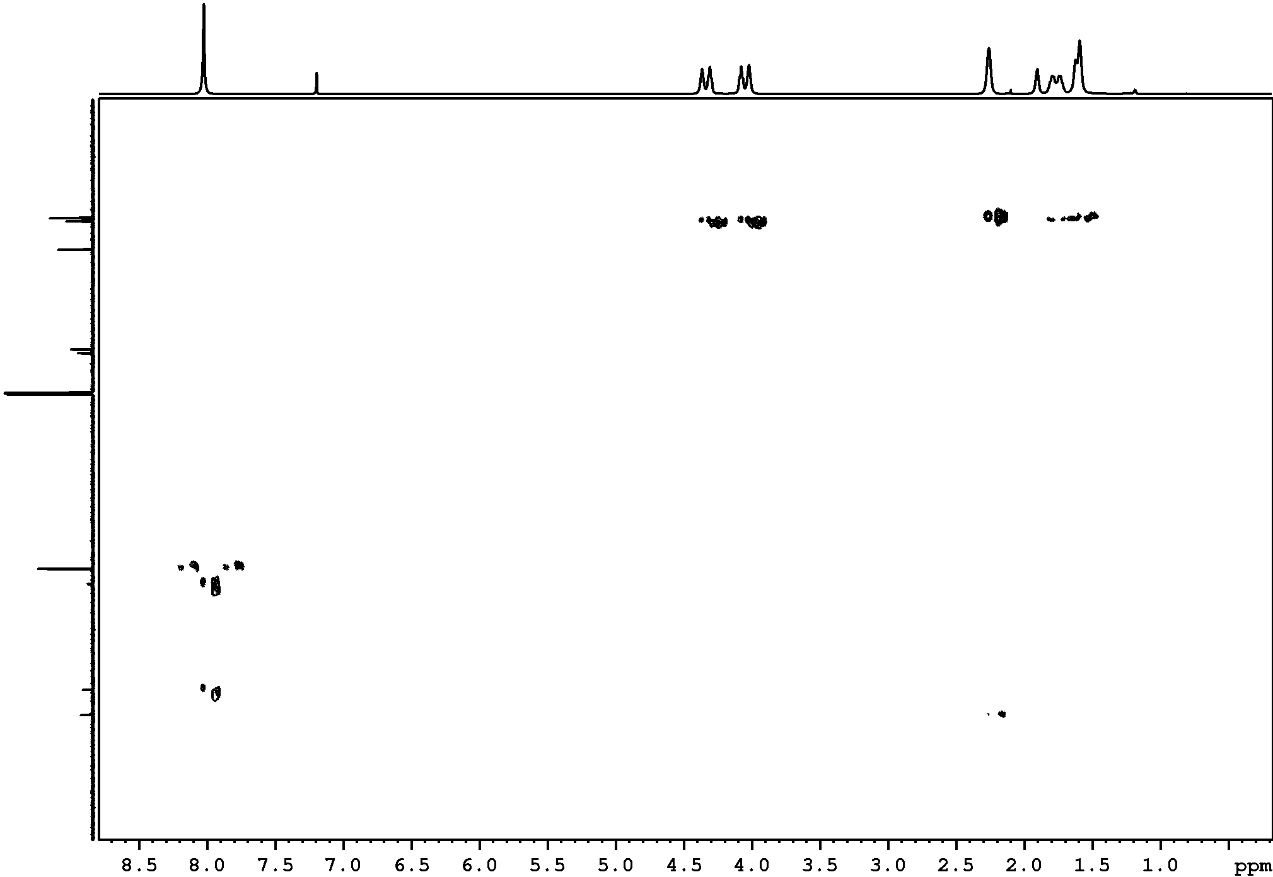


a) ^13^C NMR; b) COSY; c) Edited HSQC; d) HMBC.

# Figure S3. Ampliated region (δ = 3-8 ppm) of ^1^H NMR spectra shown in Figure 3


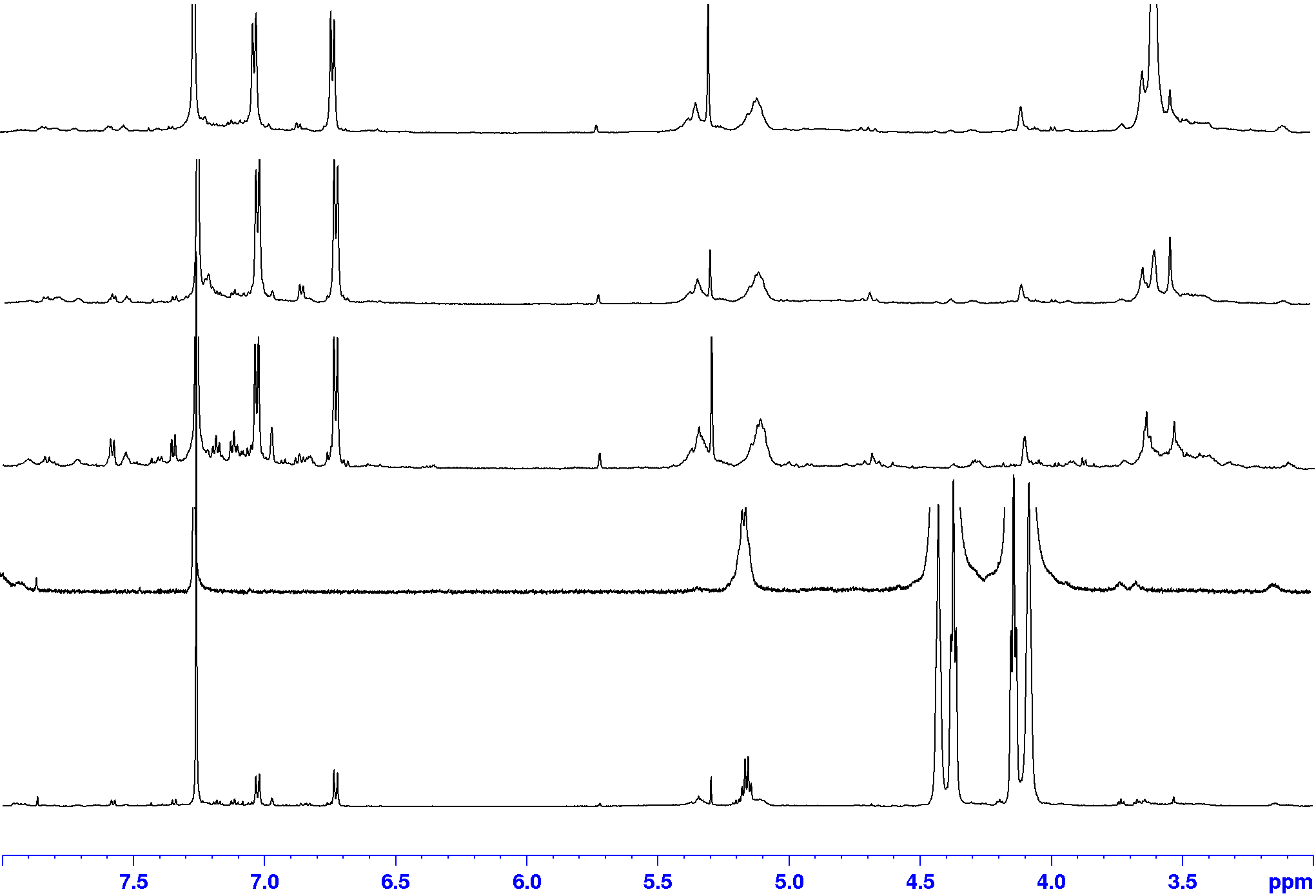


**a**

**b**

**c**

**d**

a) Inoculum from a blank reactor after a BMP assay plus 5 mg of commercial bioplastic **TB1** added to the CHCl_3_ used for its extraction (positive control); b) commercial bioplastic **TB1** (reference); c) inoculum from a blank reactor after a BMP assay (negative control); d) extracted digestate obtained after anaerobic digestion (69 days) of bioplastic **TB1** (target).

# Figure S4. Selected ATR-FTIR spectra


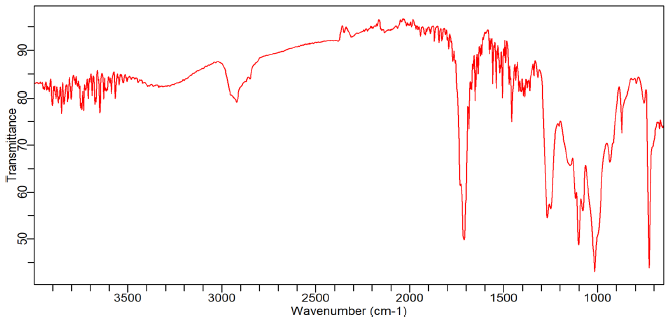


**b**


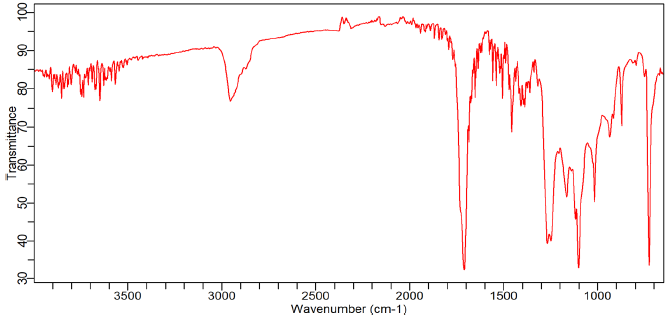

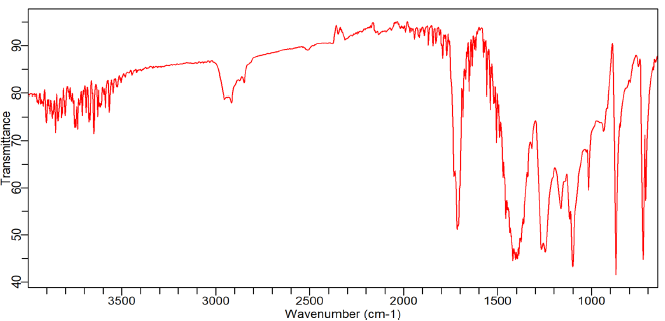

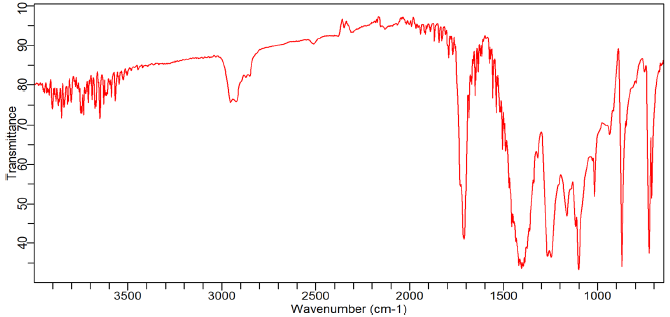


**a**

Initial (up) and final (down) ATR-FTIR spectra of **GB** (a) and **TB1** (b) bags.

# Figure S5. Selected ^1^H NMR spectra of the six studied bioplastic bags before and after the anaerobic digestion

**a**


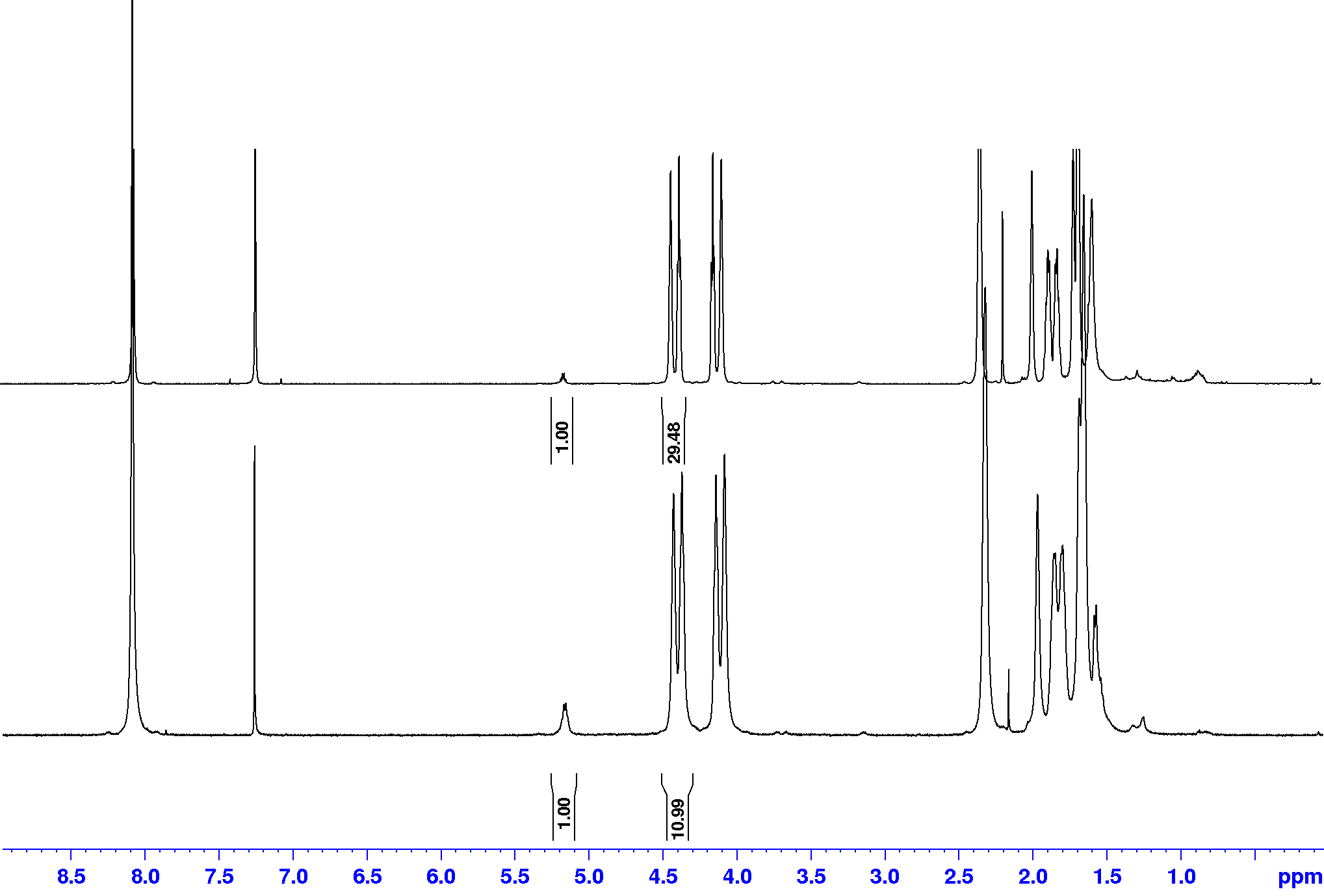

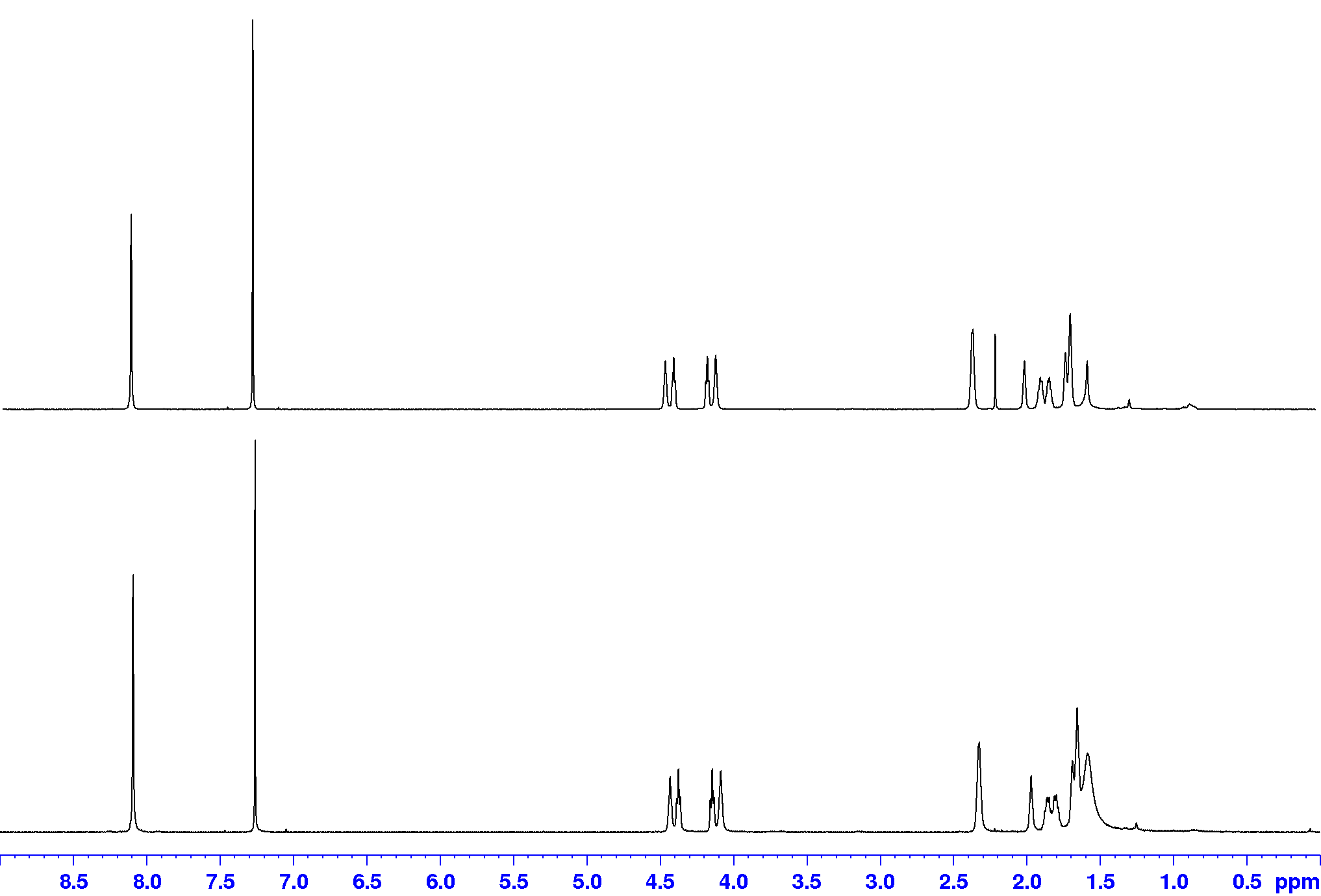


**b**

**c**


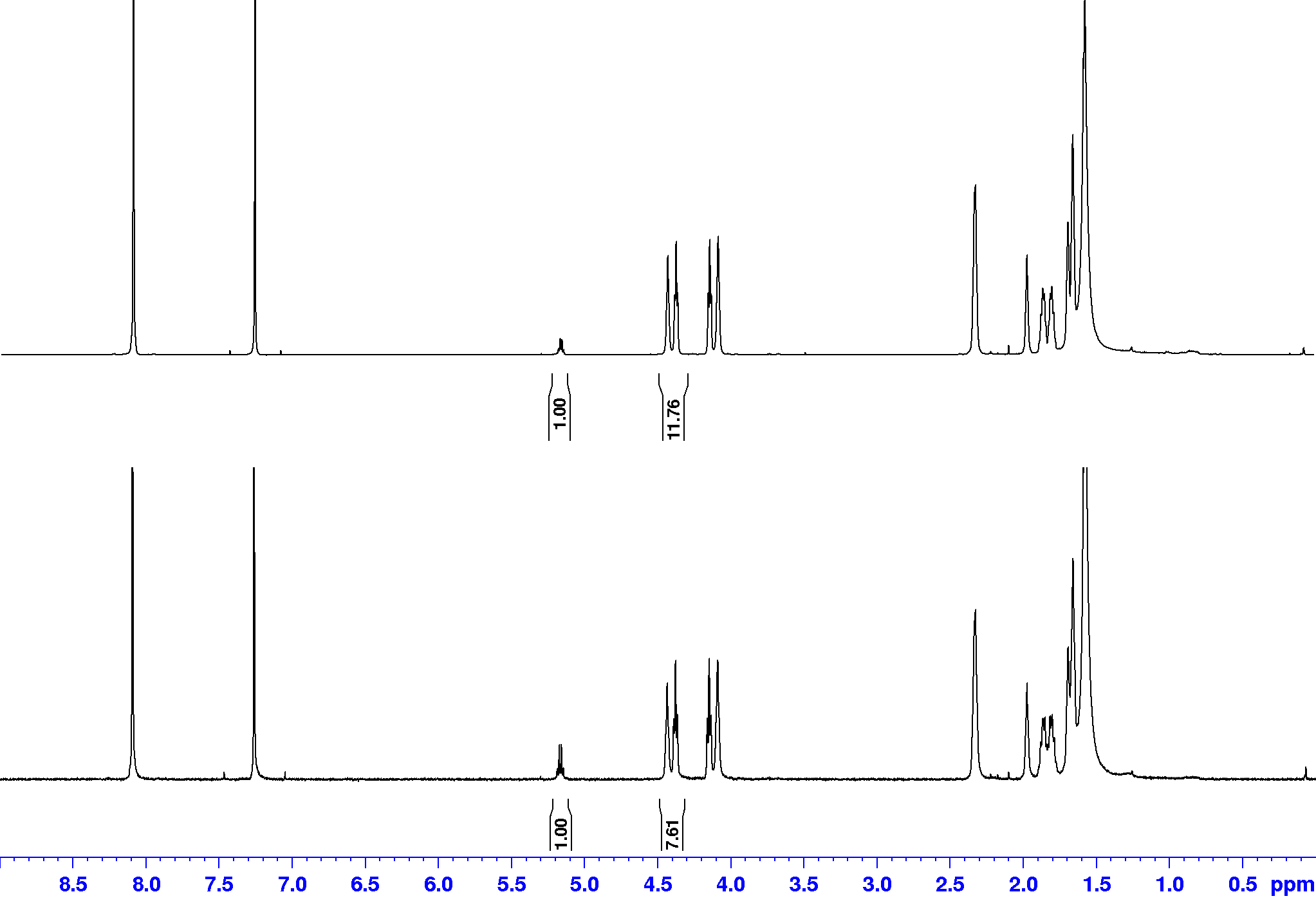

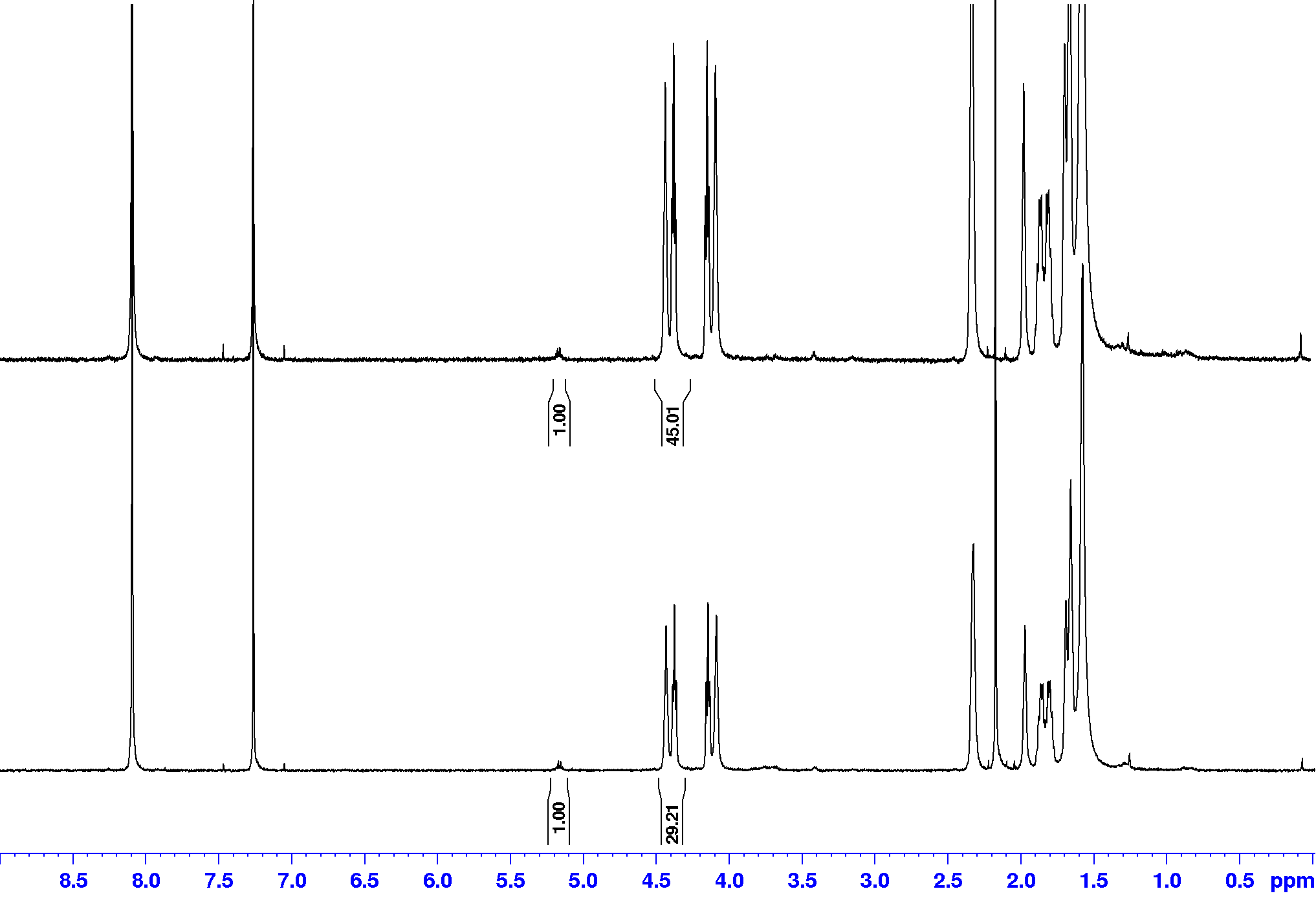


**d**


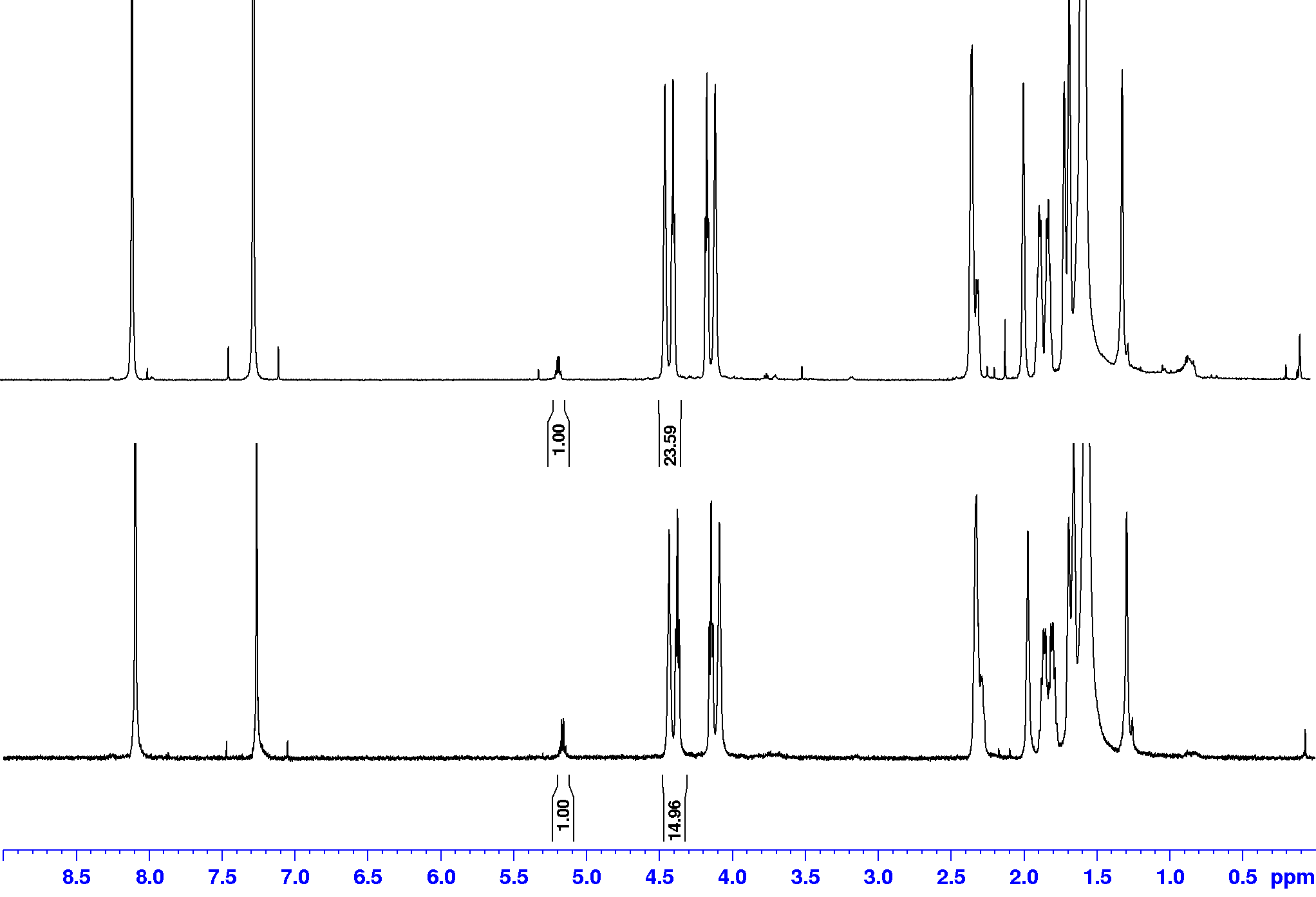


**e**

**f**


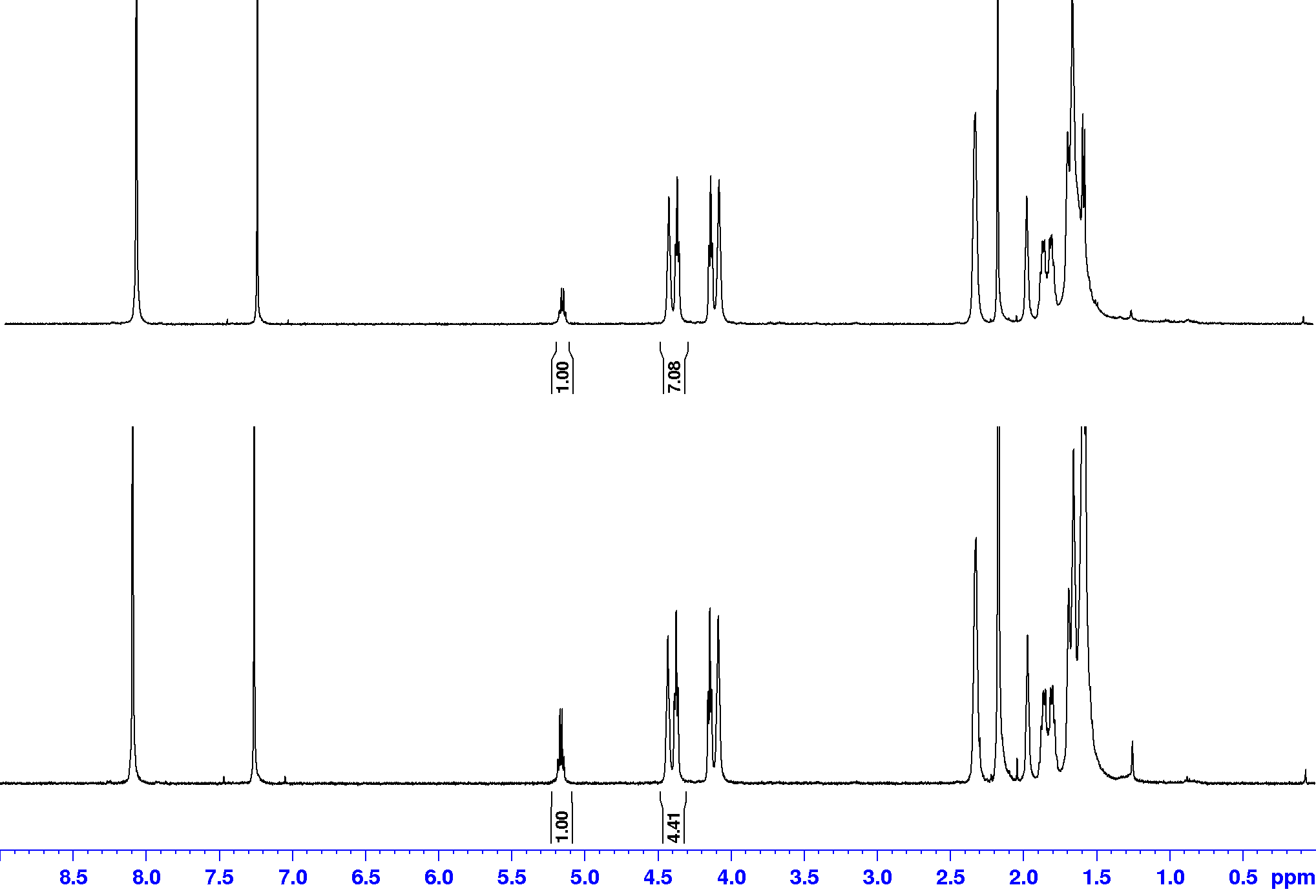


Initial (down) and final (up) ^1^H NMR spectra of **TB1** (a), **GB** (b), **TB2** (c), **FB1** (d), **FB2** (e) and **PB** (f).
